# An Atlas of Clinical Microscopy

**Published:** 1886-01

**Authors:** 


AN ATLAS OF CLINICAL MICROSCOPY. By Alexander Peyer, M. D. Translated by A. C. Girard, M. D. New York : D. Appleton & Company. 1885. 8vo., pp. 194.

The work contains 105 chromo lithographs made from original drawings, and are arranged into nine parts. Part I. Microscopic examination of the blood. II. Mammary secretion. III. Urine. IV. Sputum. V. Intestinal contents. VI. Contents of stomach. VII. Contents of abdominal tumors. VIII. Secretions of female sexual organs. IX. Micro-organisms. The plates are fair representations of the histology of the blood and various secretions under different pathological conditions, and they are accompanied by a brief incomplete explanatory text. The work is designed for physicians as an aid in diagnosis. To appreciate this work the physician must be familiar with the use of the microscope, and have some knowledge of microscopic technology and normal histology. The book will prove useful for reference, as it furnishes microscopical pictures with which specimens prepared for examination can be compared. The plates are clear, the form elements well defined, and in every way they are altogether excellent, but they do not cover the field of clinical microscopy. Under the subject of parasites of the blood the author fails to mention the *filaria sanguinis*. There exists really no good book in this line of work, and the one before us leaves much to be desired. H. W.

